# Molecular detection of *Anaplasma phagocytophilum* in field-collected *Haemaphysalis* larvae in the Republic of Korea

**DOI:** 10.1186/s13071-024-06649-z

**Published:** 2025-02-21

**Authors:** KyuSung Ahn, Badriah Alkathiri, Seung-Hun Lee, Haeseung Lee, Dongmi Kwak, Yun Sang Cho, Hyang-Sim Lee, SoYoun Youn, Mi-Sun Yoo, Jaemyung Kim, SungShik Shin

**Affiliations:** 1 BIOREEDS Research Institute, College of Veterinary Medicine, Chonnam National University, Gwangju, 61186 Republic of Korea; 2 College of Veterinary Medicine, Chungbuk National University, 1 Chungdae-ro, Cheongju, 28644 Republic of Korea; 3 College of Veterinary Medicine, Kyungpook National University, 80 Daehak-ro, Daegu, 41566 Republic of Korea; 4 Viral Disease Division, Department of Animal and Plant Health Research, Animal and Plant Quarantine Agency, Gimcheon, 39660 Republic of Korea; 5 Parasitic and Honeybee Disease Laboratory, Bacterial Disease Division, Department of Animal and Plant Health Research, Animal and Plant Quarantine Agency, Gimcheon, 39660 Republic of Korea; 6 Laboratory of Parasitology, College of Veterinary Medicine, Chonnam National University, Gwangju, 61186 Republic of Korea

**Keywords:** *Haemaphysalis longicornis*, *Anaplasma phagocytophilum*, Transovarial transmission, Larvae, Epidemiology, Tick-borne diseases

## Abstract

**Background:**

*Anaplasma* spp., zoonotic tick-borne pathogens affecting livestock, companion animals, and humans, exhibits 15–18% seropositivity among hunting dogs in the Republic of Korea (South Korea). The dominant tick species in South Korea, *Haemaphysalis longicornis*, can transmit these pathogens to both humans and animals. Given the limited understanding of transovarial transmission of *Anaplasma* spp., our study aimed to assess the prevalence of questing larval ticks containing *Anaplasma* DNA. Additionally, we aimed to gather data for establishing a nationwide forecasting and alert system on seasonal variation of tick developmental stages and tick-borne zoonotic pathogens.

**Methods:**

From March to October 2021 and again from March to October 2022, we collected a total of 36,912 unfed, questing ticks of *Haemaphysalis* spp. from 149 sites in South Korea. Ticks were collected from herbaceous vegetation using the flagging method using a white flannel cloth. After species identification, one-third of collected ticks underwent analysis for *Anaplasma* DNA. Nymph ticks were pooled in groups of 1–10 and larvae in groups of 1–50, while adults were examined individually. Nested polymerase chain reaction (PCR) was performed to detect the genus *Anaplasma* by amplifying the 16S rRNA gene, followed by sequencing for species identification and phylogenetic analysis.

**Results:**

Of the 36,912 questing ticks collected, 13,082 (35.4%) were identified as nymphs and adults of *H. longicornis* and 3850 (10.4%) as those of *Haemaphysalis flava*. The morphologically indistinguishable larval stage of *Haemaphysalis* spp. predominated, with 19,980 (54.1%) collected primarily from July to October. From the 939 tick pools, 24 pools (2.6%) tested positive for *Anaplasma*, with the larval stage exhibiting the highest number of positive pools (16, 1.7%). Phylogenetic analysis revealed that 21 of the 24 *Anaplasma*-positive pools contained *A. phagocytophilum*-specific genes, with 1 identified as *Anaplasma* sp. and the remaining 2 as *A. bovis*.

**Conclusions:**

Our study provides evidence of transovarial transmission of *A. phagocytophilum* in *Haemaphysalis* spp. larvae under field conditions, showing that the bacteria are transmitted from mother ticks to unengorged, questing larvae. Additionally, our findings contribute significant data for establishing a nationwide forecasting and alert system on seasonal variation of tick developmental stages and tick-borne zoonotic pathogens.

**Graphical Abstract:**

**Figure:**
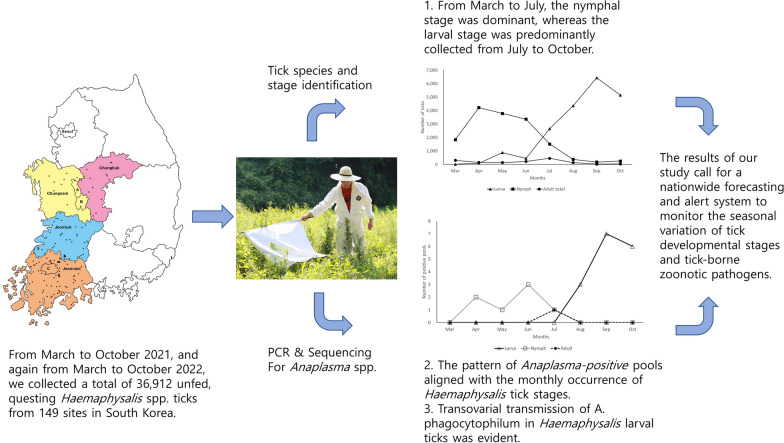

**Supplementary Information:**

The online version contains supplementary material available at 10.1186/s13071-024-06649-z.

## Background

The longhorned bush tick, *Haemaphysalis longicornis*, is native to eastern Asian countries, including the Republic of Korea (South Korea), Japan, and China. It has also established populations in Australia, New Zealand, several Pacific islands, and recently in the eastern USA [1]. *Haemaphysalis longicornis* is the predominant tick species found in both wild and domestic animals, as well as in humans in South Korea, followed by *Haemaphysalis flava* [2–5]. These two tick species are known to host and transmit a variety of pathogens, including *Anaplasma phagocytophilum*, *Anaplasma bovis*, *Anaplasma platys*, *Ehrlichia chaffeensis*, *Francisella tularensis*, *Bartonella henselae*, *Bartonella quintana*, *Rickettsia japonica*, *Rickettsia rickettsii*, tick-borne encephalitis virus, severe fever with thrombocytopenia syndrome virus (SFTSV), and a number of *Babesia* and *Theileria* species in South Korea [6, 7].

Among tick-borne pathogens, *A. phagocytophilum* is a particularly significant zoonotic pathogen, which infects granulocytic white blood cells in mammalian hosts and causes human granulocytic anaplasmosis [6]. A serological survey indicates that 15.6–18.8% of outdoor dogs are exposed to this pathogen in South Korea [8, 9]. *Anaplasma phagocytophilum* causes an acute febrile illness in dogs, characterized by lethargy and inappetence [10]. Less frequent signs in dogs include lameness, coughing, polydipsia, intermittent vomiting, and hemorrhages. Pathogens such as *A. phagocytophilum* in unfed larval ticks are inherited from their female mother tick through the transovarial route in *Ixodes ricinus* [11]. The larval stage of *Haemaphysalis* spp. measures only 0.58–0.62 mm in length and 0.47–0.51 mm in breadth [12], and they quest in large numbers near their hatching sites. Consequently, infestations with larval ticks often go unnoticed, while simultaneously exposing hosts to a variety of tick-borne pathogens.

This study focuses on the occurrence of *A. phagocytophilum* in *Haemaphysalis* ticks, with particular interest in the questing larval stage. Ticks were collected from four southwestern provinces of South Korea, and *Anaplasma*-positive *Haemaphysalis* ticks were identified and analyzed.

## Materials and methods

### Tick collection

**Fig. 1:**
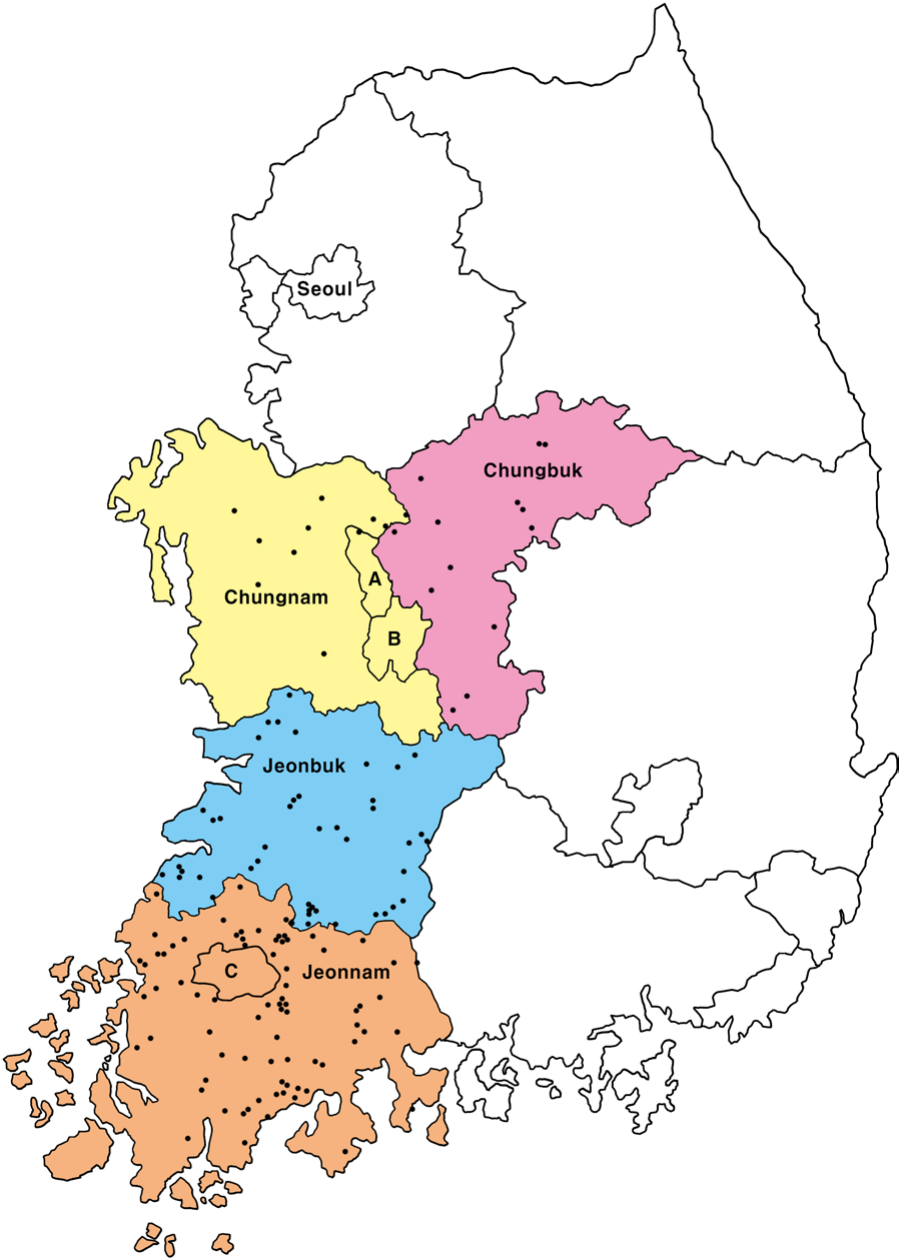
Map for the tick collection sites of the four southwestern regions in the mainland of the Republic of Korea (South Korea). Each dot represents 1 of 149 collection sites. **A** Sejong Special Self-Governing City, **B** Daejeon Metropolitan City, **C** Gwangju Metropolitan City

Unfed, questing ticks were collected from herbaceous vegetation using the flagging method, employing a white flannel cloth measuring 100 × 100 cm. Ticks were collected from 149 sites across the four southwestern and central provinces of South Korea between March and October 2021, and again from March to October 2022 (Fig. 1). The collection sites were located in the Jeonnam, Jeonbuk, Chungnam, and Chungbuk regions, with Daejeon Metropolitan City and Sejong Special Self-Governing City included in the Chungnam region, and Gwangju Metropolitan City included in the Jeonnam region. This study was conducted as part of a pioneering project before an annual nationwide surveillance program for tick and tick-borne diseases is launched. The study regions included areas where 18.8% of outdoor dogs tested serologically positive for *A. phagocytophilum* [9]. Collected ticks were preserved in 70% ethanol, and their developmental stages and species were identified on the basis of morphological characteristics using a stereo microscope and taxonomic key [13].

After species identification, ticks were pooled by species, developmental stage, survey period, and collection site. Adults and nymphs were identified to the species level, whereas larvae were identified to the genus level due to morphological similarities among species [13]. The number of ticks per pool ranged from 1 to 10 for nymphs and 1 to 50 for larvae, while adults were individually examined.

### DNA extraction and PCR detection

Genomic DNA was extracted using a DNeasy Blood & Tissue Kit (Qiagen, Hilden, Germany) following the manufacturer’s instructions. PCR was performed using an AccuPower HotStart PCR Premix Kit (Bioneer, Daejeon, South Korea). Nested PCR was conducted to detect the genus *Anaplasma* by amplifying the 16S rRNA gene, as previously described [14], using the primer pairs EE1/EE2 and EE3/EE4, which generated an amplicon of 924–926 bp. A sample of *A. phagocytophilum* detected in cattle in South Korea [15] served as a positive control, while a sample without a DNA template was used as a negative control. The minimum infection rate (MIR) of pooled ticks with *Anaplasma* spp. was calculated using the following equation: (number of positive pools of ticks/total number of ticks tested) × 100.

### Sequencing and phylogenetic analyses

All PCR-positive products with EE3/EE4 primers were sent to Macrogen (Daejeon, South Korea) for Sanger sequencing. The obtained sequences, along with those previously reported in GenBank, were aligned and analyzed using the multiple sequence alignment program CLUSTAL Omega (v. 1.2.1, Bioweb, Ferndale, WA, USA). Regions of poor sequence quality at the front and back ends were trimmed on the basis of the sequences detected in this study, using BioEdit (v. 7.2.5, Bioedit, Manchester, UK). Sites containing gaps or ambiguous alignment were removed before phylogenetic analysis. Phylogenetic analysis was conducted using the maximum likelihood method with the Kimura two-parameter distance model in molecular evolutionary genetics analysis [16]. Pairwise comparisons of aligned *Anaplasma* 16S rRNA gene sequences was performed to determine homology, and the stability of the obtained trees was estimated using bootstrap analysis with 1000 replicates.

For the estimation of evolutionary divergence between sequences of *Anaplasma* spp. identified in this study, analyses were conducted using the maximum composite likelihood model [17]. This analysis involved 25 nucleotide sequences, and all ambiguous positions were removed for each sequence pair (pairwise deletion option). There was a total of 881 positions in the final dataset. Evolutionary analyses were conducted in MEGA11 [16].

## Results

A total of 36,912 questing ticks of the genus *Haemaphysalis* were collected from 149 sites (Table 1). The distribution of ticks was as follows: 12,869 from Jeonnam, 5899 from Jeonbuk, 10,584 from Chungnam, and 8560 from Chungbuk provinces. Among these, larvae represented the highest proportion among the three developmental stages (19,980 ticks, 54.1%), while adults made up the lowest (1434 ticks, 3.9%). However, species identification of larvae was not recorded because the larval stages of *H. longicornis* and *H. flava* are morphologically indistinguishable.

**Table 1:** Number of *Haemaphysalis* tick species collected from the study area

Species	Stage	Number of ticks collected per province (%)				
		Jeonnam	Jeonbuk	Chungnam	Chungbuk	Total
*Haemaphysalis* spp.	Larva	7699 (20.9)	2019 (5.5)	5998 (16.2)	4264 (11.6)	19,980 (54.1)
*Haemaphysalis longicornis*	Nymph	3011 (8.2)	2297 (6.2)	4090 (11.1)	2791 (7.6)	12,189 (33.0)
	Female	261 (0.7)	77 (0.2)	283 (0.8)	97 (0.3)	718 (1.9)
	Male	35 (0.1)	18 (0.0)	95 (0.3)	27 (0.1)	175 (0.5)
Subtotal		3307 (9.0)	2392 (6.5)	4468 (12.1)	2915 (7.9)	13,082 (35.4)
*Haemaphysalis flava*	Nymph	1591 (4.3)	1265 (3.4)	109 (0.3)	344 (0.9)	3309 (9.0)
	Female	170 (0.5)	105 (0.3)	4 (0.0)	10 (0.0)	289 (0.8)
	Male	102 (0.3)	118 (0.3)	5 (0.0)	27 (0.1)	252 (0.7)
Subtotal		1863 (5.0)	1488 (4.0)	118 (0.3)	381 (1.0)	3850 (10.4)
Combined	Larva	7699 (20.9)	2019 (5.5)	5998 (16.2)	4264 (11.6)	19,980 (54.1)
	Nymph	4602 (12.5)	3562 (9.6)	4199 (11.4)	3135 (8.5)	15,498 (42.0)
	Adult	568 (1.5)	318 (0.9)	387 (1.0)	161 (0.4)	1434 (3.9)
Total		12,869 (34.9)	5899 (16.0)	10,584 (28.7)	7560 (20.5)	36,912 (100.0)

Nymph and adult stages of *H. longicornis* (13,082 ticks, 35.4%) and *H. flava* (3850 ticks, 10.4%) were collected from all four provinces, with *H. longicornis* being the dominant species. The occurrence of *H. longicornis* nymphs and adults was primarily observed from April to July, with peak occurrences in May for nymphs and July for adults, respectively. In contrast, the occurrence of *H. flava* nymphs and adults was observed from March to June, with peak occurrence in March for both nymphs and adults (Additional file 1: Table S1).

**Fig. 2:**
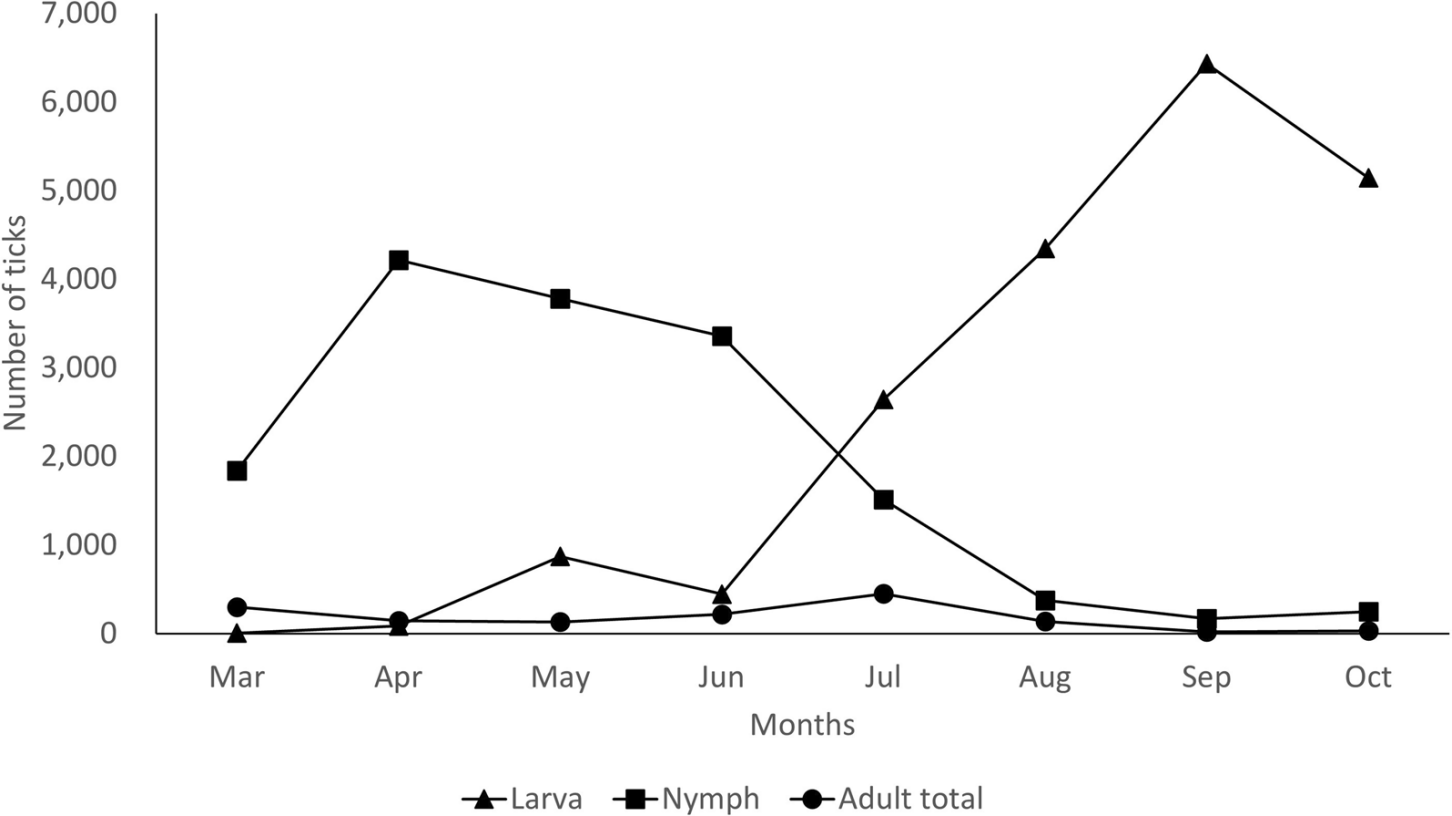
Monthly occurrence of *Haemaphysalis* ticks by developmental stages

The monthly occurrence of *Haemaphysalis* ticks by developmental stages from March to October is illustrated in Fig. 2, with a detailed breakdown presented in Additional file 1: Table S1. From March to July, the nymphal stage dominated the study areas, with 94.9% of the nymphal ticks collected during this period, peaking in April. Conversely, the larval stage was predominantly collected from July to October, accounting for 92.9% of the larvae collected during these months, with the highest peak observed in September. The collection of adult stages was comparatively low (1434 ticks, 3.9%) compared with the larval (19,980 ticks, 54.1%) and nymphal (15,498 ticks, 42.0%) stages, with the highest number of adults collected in July (451 ticks, 1.3%).

Of the ticks collected, approximately one-third (13,118 ticks, 35.5%) underwent PCR analysis to detect *Anaplasma* infection. They were pooled into 939 groups, which were then subjected to PCR analysis followed by sequencing for species identification and phylogenetic analysis. Out of the 939 pools of ticks, 24 pools (2.6%) tested positive for *Anaplasma*. The larval stage exhibited the highest number of *Anaplasma*-positive pools (16 pools, 1.7%), followed by the nymphal (7 pools, 0.7%) and adult (1 pool, 0.1%) stages (Table 2).

**Table 2:** Source of *Anaplasma*-positive tick pools of *Haemaphysalis* spp. in Korea

Species	Stage	Number of *Anaplasma*-positive pools (%)					No of ticks	Number of	MIR within	MIR^1^
		Jeonnam	Jeonbuk	Chungnam	Chungbuk	Total	for PCR (%)	PCR pools (%)	Stage	
*Haemaphysalis* spp.	Larva	5 (0.53)	1 (0.11)	4 (0.43)	6 (0.64)	16 (1.70)	7153 (19.4)	156 (16.6)	0.224	0.122
*Haemaphysalis longicornis*	Nymph	0 (0.00)	0 (0.00)	4 (0.43)	3 (0.32)	7 (0.75)	4441 (12.0)	461 (49.1)	0.158	0.053
	Female	0 (0.00)	0 (0.00)	0 (0.00)	0 (0.00)	0 (0.00)	106 (0.3)	106 (11.3)	0.000	0.000
	Male	0 (0.00)	0 (0.00)	1 (0.11)	0 (0.00)	1 (0.11)	25 (0.1)	25 (2.7)	4.000	0.008
Subtotal		0 (0.00)	0 (0.00)	5 (0.53)	3 (0.32)	8 (0.85)	4572 (12.4)	592 (63.0)	0.175	0.061
*Haemaphysalis flava*	Nymph	0 (0.00)	0 (0.00)	0 (0.00)	0 (0.00)	0 (0.00)	1355 (3.7)	153 (16.3)	0.000	0.000
	Female	0 (0.00)	0 (0.00)	0 (0.00)	0 (0.00)	0 (0.00)	19 (0.1)	19 (2.0)	0.000	0.000
	Male	0 (0.00)	0 (0.00)	0 (0.00)	0 (0.00)	0 (0.00)	19 (0.1)	19 (2.0)	0.000	0.000
Subtotal		0 (0.00)	0 (0.00)	0 (0.00)	0 (0.00)	0 (0.00)	1393 (3.8)	191 (20.3)	0.000	0.000
Combined	Larva	5 (0.53)	1 (0.11)	4 (0.43)	6 (0.64)	16 (1.70)	7153 (19.4)	156 (16.6)	0.224	0.122
	Nymph	0 (0.00)	0 (0.00)	4 (0.43)	3 (0.32)	7 (0.75)	5796 (15.7)	614 (65.4)	0.121	0.053
	Adult total	0 (0.00)	0 (0.00)	1 (0.11)	0 (0.00)	1 (0.11)	169 (0.5)	169 (18.0)	0.592	0.008
Total		5 (0.53)	1 (0.11)	9 (0.96)	9 (0.96)	24 (2.56)	13,118 (35.5)	939 (100.0)	0.183	0.183

^1^ *MIR* minimum infection rate (%)

*Anaplasma*-positive pools were found in the nymphal and adult stages of *H. longicornis*, but none of the *H. flava* pools contained *Anaplasma*-specific DNA. The minimum infection rate (MIR) of *Anaplasma* from the 13,118 ticks was calculated to be 0.183%. High numbers of *Anaplasma*-positive pools were observed in Chungnam and Chungbuk provinces, each with nine positive pools, while Jeonnam and Jeonbuk provinces had five and one positive pools, respectively.

**Fig. 3:**
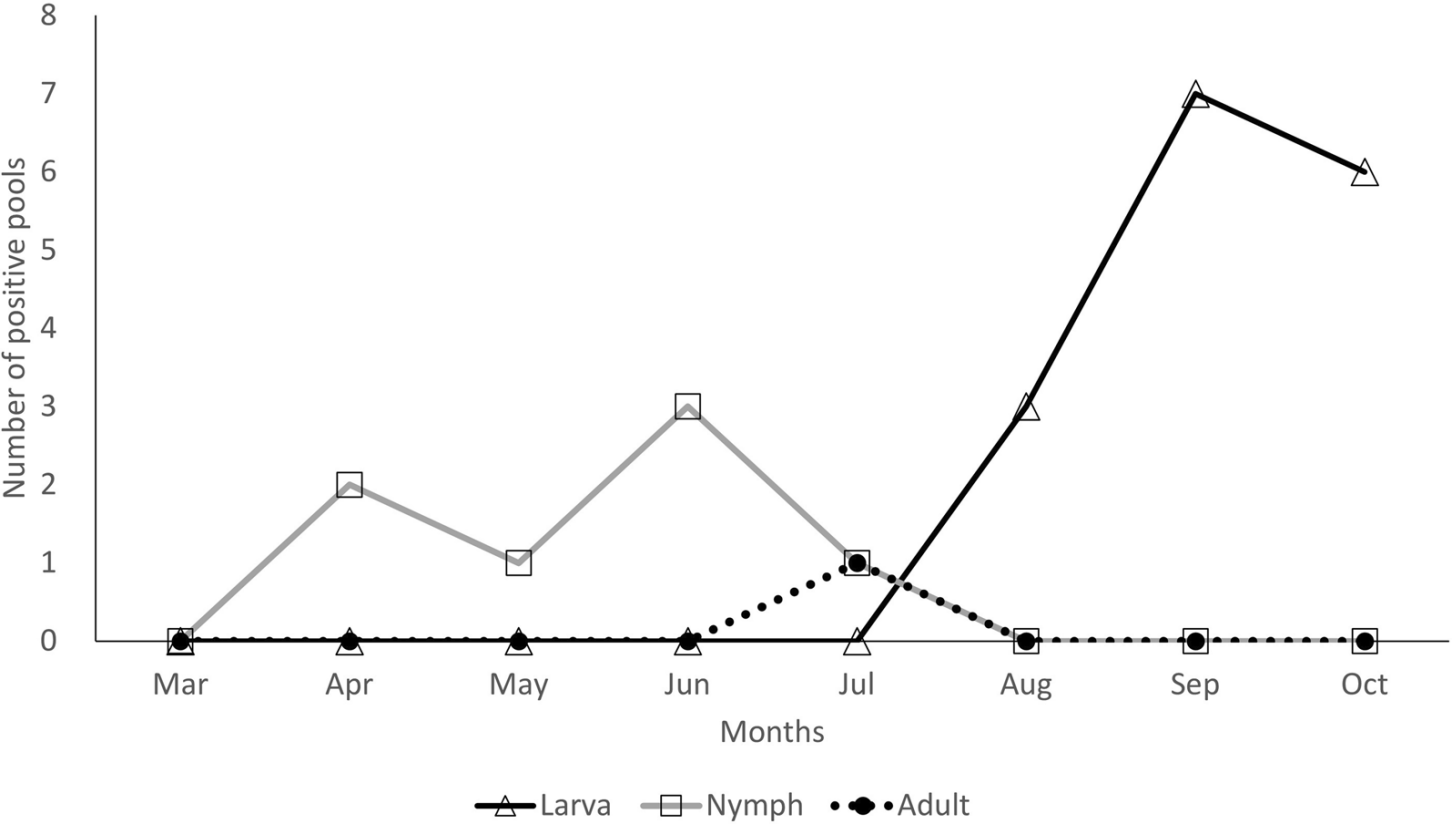
Monthly occurrence of *Anaplasma* spp. in *Haemaphysalis* ticks by developmental stages

The monthly occurrence of *Anaplasma*-positive pools is depicted in Fig. 3. The pattern of *Anaplasma*-positive pools coincided with the monthly occurrence of *Haemaphysalis* tick stages. Positive pools in the nymphal stage were found from April to July, while *Anaplasma*-positive larval pools were observed from August to October. One positive pool in the adult stage was found in July, coinciding with the peak occurrence of adult ticks. The highest peak occurrence of *Anaplasma*-positive pools was observed in September, which corresponded with the peak in larval tick activity.

**Fig. 4:**
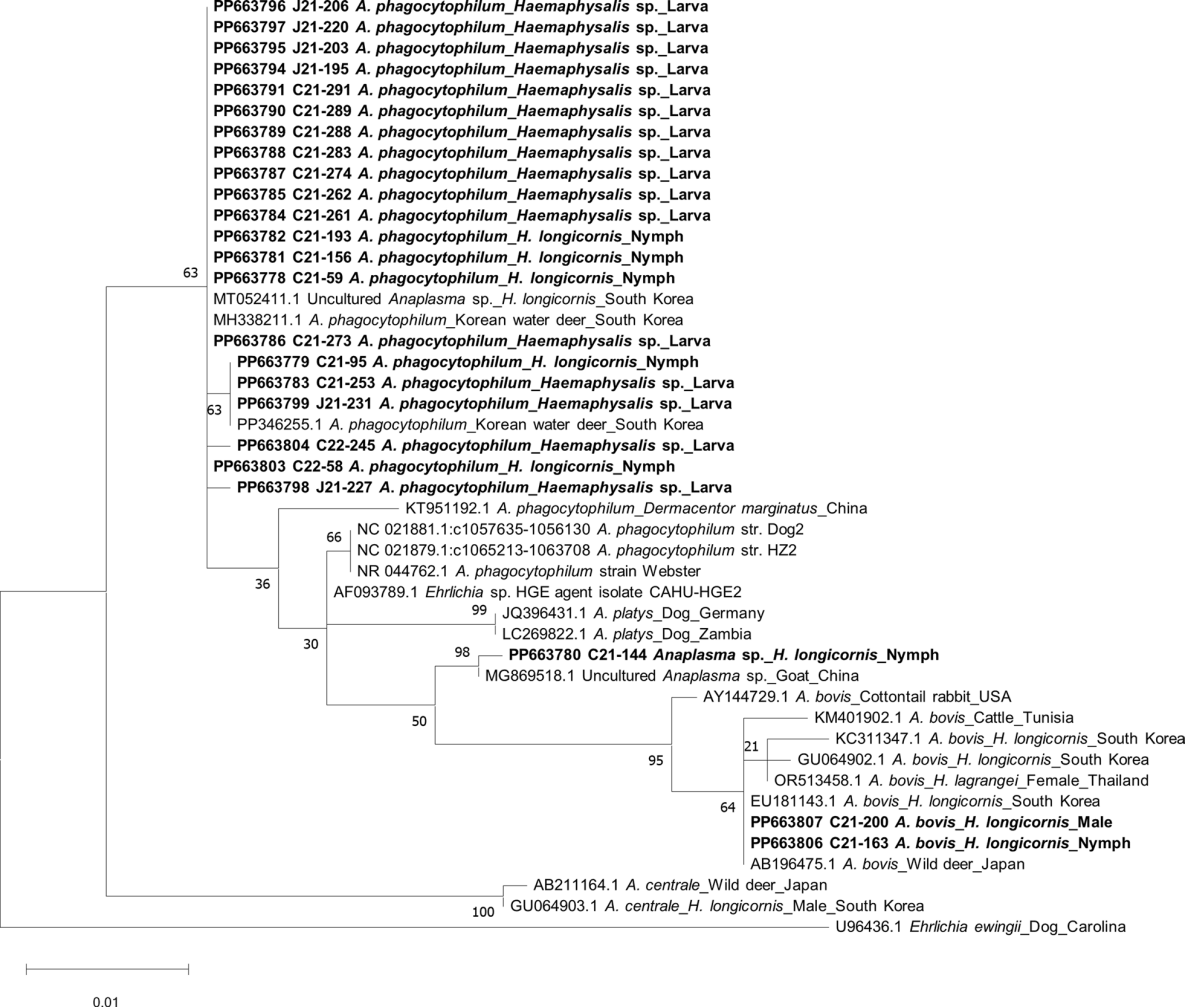
Phylogenetic tree of *Anaplasma phagocytophilum* based on sequences of the 16S rRNA gene. The tree was constructed using the maximum likelihood method with the Kimura two-parameter distance model. Sequences in bold fonts represent those in the present study. GenBank accession numbers of other sequences presented with the sequence name. Numbers on the branches correspond to mean bootstrap support at 1000 replicates. The scale and the scale bar show phylogenetic distance

Phylogenetic analysis of the PCR-positive pools for *Anaplasma* spp. revealed that 21 of the 24 *Anaplasma*-positive pools contained *A. phagocytophilum*-specific gene, while the remaining 2 and 1 were identified as *A. bovis* and *Anaplasma* sp., respectively (Fig. 4). The 21 *A. phagocytophilum*-specific sequences were closely clustered together, regardless of the regions of tick collection within South Korea, and were closely aligned with previously reported sequences of *A. phagocytophilum* isolated from water deer in South Korea. However, they clustered separately and were distantly related to previously reported *A. phagocytophilum* reference strains such as Webster, HGE2, HZ, and Dog2 (Fig. 4).

The two *A. bovis* sequences were found in a pool of *H. longicornis* nymphs collected in Sejong-si in June and in an adult male tick collected in Chungnam province in July. A total of 16 *A. phagocytophilum*-positive pools were identified from larval ticks, while 6 positive pools were from nymphs.

The mean divergence between the 16 sequences of *A. phagocytophilum* from the larval stage was 0.0021 ± 0.0048 (mean ± SD, max 0.0207, Additional file 1: Table S2). A total of 21 sequences of *A. phagocytophilum* identified in this study, including all 16 sequences from larval ticks, were grouped together (Fig. 4). The mean divergence of the 24 *A. phagocytophilum* sequences compared with *A. phagocytophilum* identified from a Korean water deer was 0.0033 ± 0.0077. Therefore, *A. phagocytophilum* identified from the 16 larval pools of *Haemaphysalis* ticks appeared to be highly similar. The 16 larval pools of *A. phagocytophilum* originated from all four provinces investigated in this study: 5 from Jeonnam, 1 from Jeonbuk, 4 from Chungnam, and 6 from Chungbuk.

Representative sequences obtained from this study and used in the phylogenetic analysis were submitted to the GenBank database (accession numbers: PP663778–PP663807).

## Discussion

### Seasonal occurrence of *Haemaphysalis* ticks in South Korea compared with other regions

The survey conducted in the southwestern provinces of South Korea during 2021 and 2022 revealed *H. longicornis* as the predominant tick species, followed by *H. flava*. Nymphal ticks were prevalent from March to July, while larval ticks were most commonly collected from July to October, with adult ticks peaking in July. These findings are consistent with previous reports on the seasonal distribution of *H. longicornis* near the demilitarized zone in South Korea and in Virginia, USA [18, 19]. The life cycle of *H. longicornis* in these regions follows a similar seasonal trend, with adults active in the summer, larvae peaking in the fall, and nymphs overwintering and becoming active in the spring [20–22]. Given that *H. longicornis* is associated with large wild and domestic mammals and *H. flava* with small- to medium-sized mammals and birds [18], Korea’s landscape of grass- and herb-covered hills with scattered trees provides an ideal habitat for both species.

### Larval ticks as the major source for *Anaplasma* transmission

Results of this study indicates that the larval stage of *Haemaphysalis* ticks carried significantly more *Anaplasma* pathogens than the nymphal or adult stages, as two-thirds of *Anaplasma*-positive pools were from larval ticks. The high occurrence of *Anaplasma*-positive larvae likely stems from the large number of larvae collected. The minimum infection rate (MIR) for the larval stage was 0.224, compared with 0.121 for nymphs and 0.592 for adults (Table 2). Although the adult stage had a higher MIR, the large number of larvae and the presence of transovarial transmission of *A. phagocytophilum* in *Haemaphysalis* ticks suggest that larval ticks are an important vector for *A. phagocytophilum* in South Korea.

### MIR of *A. phagocytophilum* in different tick stages

The lower MIR in nymphal ticks compared with larval ticks may be influenced by various factors. While a higher number of positive pools in nymphal ticks might be expected due to the cumulative effect of transovarial transmission and additional new infections during blood-feeding in the larval stage, the host preferences of different tick stages and the infection status of *A. phagocytophilum* in these hosts could affect the infection rates. Although previous studies have found *A. phagocytophilum* in adult *H. longicornis* in South Korea [23], *A. phagocytophilum* was not detected in adult *H. longicornis* in this study. The small sample size of adult ticks (169 compared with 7153 larvae for PCR) may explain this result.

### Variation of *A. phagocytophilum* genes isolated from field *Haemaphysalis* ticks

This study showed that the 21 *A. phagocytophilum*-specific sequences identified from *Haemaphysalis* ticks were closely clustered together (Fig. 4). However, they were somewhat distantly aligned with previously reported reference strains of *A. phagocytophilum* isolated from either humans (HZ, HGE2, Webster) or dogs (Dog2) [24–27]. Since the 21 *A. phagocytophilum*-specific sequences were isolated from ticks collected from herbaceous vegetation using the flagging method and not from humans or dogs, it is likely that the origin of the 21 *A. phagocytophilum* is primarily from wildlife animals.

### Fall as the larval tick season in South Korea

*Anaplasma*-positive larval ticks were observed from July to October, with a peak in September (Fig. 3). This suggests that public health campaigns in South Korea should emphasize the increased risk of *Anaplasma* transmission during this period, particularly from the tiny, difficult-to-detect larval ticks, which measure only 0.6 mm in length [12]. The increased risk of exposure to *Anaplasma* infection during fall in South Korea is similar to what is observed in scrub typhus transmission in the country. Scrub typhus, also known as tsutsugamushi disease, is a common febrile vector-borne disease in South Korea caused by an intracellular bacterium, *Orientia tsutsugamushi* [28]. The disease is transmitted by the chigger, the larval stage of the trombiculid mite, and has a marked seasonality in incidence, peaking in autumn [29]. Anaplasmosis and scrub typhus would therefore better be addressed together during outdoor activities in the autumn season in South Korea.

### The importance of transovarial transmission for *A. phagocytophilum*

This study strongly supports the transovarial transmission of *A. phagocytophilum* from female *Haemaphysalis* ticks to their larvae, as all collected larvae were questing, unengorged ticks from vegetation rather than host animals. This finding is consistent with studies on *Ixodes ricinus* in Germany [11] and *Dermacentor albipictus* in North America [30]. While *A. phagocytophilum* was the predominant *Anaplasma* species detected, *A. bovis* was also found in one nymphal and one adult tick pool, highlighting the veterinary and medical significance of transovarial transmission in tick-borne diseases.

### High prevalence of *A. phagocytophilum* in dogs in South Korea and larval tick infestation

The role of larval *Haemaphysalis* ticks in transmitting *Anaplasma* to human or animal hosts has not been fully investigated, and there are no reports of clinical cases of anaplasmosis caused by larval ticks in South Korea. However, previous studies have reported a relatively high seroprevalence of *A. phagocytophilum* (15.6–18.8%) among outdoor dogs, including hunting dogs, in South Korea [8, 9]. Although no specific data on the contribution of larval ticks to this high seroprevalence were provided, the tiny size of larval *Haemaphysalis* ticks and their abundant distribution may explain the high infection rates in dogs. One of the authors in this study has created a YouTube video demonstrating the potential dangers of larval tick infestations on dogs in South Korea [31].

### Forecasting and public awareness campaigns on larval ticks

Given the high prevalence of *Anaplasma* spp. in questing larval ticks, it appears to be crucial to raise awareness about the risks of tick-borne infections for both animals and humans by larval ticks. Preventive measures should be emphasized, especially for long-haired animals, as larval ticks are difficult to detect. The high tick occurrence throughout the spring–summer–fall seasons, as indicated in Fig. 2, underscores the need for forecasting and monitoring tick populations and pathogens. Public health campaigns, similar to those for mosquito-borne diseases like Japanese encephalitis, should be implemented to mitigate the risk of tick-borne illnesses in South Korea [32].

## Conclusions

This study provides valuable insights into the prevalence and transmission dynamics of *Anaplasma* spp. in *Haemaphysalis* ticks in South Korea. The findings highlight the importance of transovarial transmission in the epidemiology of tick-borne pathogens and underscore the need for proactive measures to mitigate the risk of tick-borne diseases. Additionally, we recommend implementing a nationwide forecasting and alert system to monitor seasonal variations in tick developmental stages and tick-borne zoonotic pathogens such as *A. phagocytophilum*.

## Supplementary Information

Additional file 1.

## Data Availability

No datasets were generated or analyzed during the current study.

## Abbreviations

MIR: Minimum infection rate
s.l.: Sensu lato
TBE: Tick-borne encephalitis
